# Oral Bioavailability of a Noncoding RNA Drug, TY1, That Acts on Macrophages

**DOI:** 10.1002/jex2.70081

**Published:** 2025-08-14

**Authors:** Shukuro Yamaguchi, Kazutaka Miyamoto, Xaviar M. Jones, Alessandra Ciullo, Kara Tsi, Jessica Anderson, Hiroaki Komuro, Salwa Soussi, Ashley Morris, Diana Kitka, De‐Zhao Liu, Anh Nguyen, Eduardo Marbán, Ahmed G. E. Ibrahim

**Affiliations:** ^1^ Smidt Heart Institute Cedars‐Sinai Medical Center Los Angeles California USA

**Keywords:** acute lung injury, biodistribution, hypertrophic cardiomyopathy, macrophages, myocardial infarction, oral formulation, small intestine, small RNA

## Abstract

All approved RNA therapeutics require parenteral delivery. Here, we demonstrate an orally bioavailable formulation wherein synthetic noncoding (nc) RNA, packaged into lipid nanoparticles, is loaded into casein‐chitosan (C2) micelles. We used the C2 formulation to deliver TY1, a 24‐nucleotide synthetic ncRNA, which targets DNA damage and attenuates inflammation in macrophages. C2‐formulated TY1 (TY1^C2^) efficiently packages and protects TY1 against degradative enzymes. In healthy mice, oral TY1^C2^ was well‐tolerated and nontoxic. Oral TY1^C2^ exhibited disease‐modifying bioactivity in two models of tissue injury: (1) rat myocardial infarction, where a single oral dose of TY1^C2^ was cardioprotective, on par with intravenously‐delivered TY1; and (2) mouse acute lung injury, where a single dose of TY1^C2^ attenuated pulmonary inflammation. Mechanistic dissection revealed that TY1^C2^ is taken up by intestinal macrophages, namely those of the lamina propria and Peyer's patches. Afterwards, TY1 could be detected in circulating monocytes for up to 72 h post‐ingestion. Unlike TY1, which acts on macrophages, an antisense oligonucleotide against Factor VII, which acts on hepatocytes, is not effective when administered in the C2 formulation. Thus, not all ncRNA drugs are bioactive when delivered by mouth. Oral delivery of macrophage‐active RNA opens up a wide range of potential new therapeutic opportunities.

## Graphical abstract

Oral TY1^C2^ is taken up in the small intestine by macrophages of the lamina propria and the Peyer's patches. TY1 later is detectable in circulating mononuclear cells, namely monocytes. In a rat model of MI, oral TY1^C2^ leads to cardioprotection and pulmonary protection.

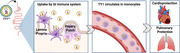

## 1 Introduction

RNA drugs have immense potential to impact both conventional as well as novel therapeutic targets, but they face a significant hurdle: delivery. RNA drug delivery has to be effective without triggering an undesired immune reaction. Parenteral lipid nanoparticle (LNP)‐packaged RNA has met with some success, but immunogenicity remains a concern (Kaczmarek et al. 2017; Qin et al. 2022). Systemic intravenous infusion, while less invasive than most other parenteral routes, is problematic, especially if frequent dosing is required (Jin et al. 2015). Subcutaneous delivery of naked antisense oligonucleotides can work quite well when targeting the liver, but not for other organs (Witten et al. 2024). A means to deliver RNA drugs orally, if safe and effective, is highly desirable (Forbes and Peppas 2012), especially for chronic disorders. Oral delivery that would effectively target tissues other than the liver would be even more desirable. However, the efficient oral delivery of RNA remains challenging, given the harsh physicochemical environment of the gastrointestinal (GI) tract: highly acidic, nuclease‐rich and coated by a mucin barrier. We have created TY1, a 24 nucleotide chemically stabilised ncRNA drug that, when given IV, reduces tissue injury in models of myocardial infarction (MI) (Ibrahim et al. 2024). Here, we describe a new, simple oral formulation that not only replicates the effects of intravenous TY1 on MI but also exhibits disease‐modifying bioactivity in two other disease models (one shown here, and another described earlier (Miyamoto et al. 2024)).

## 2 Results

### 2.1 Casein‐Chitosan Formulation Efficiently Encapsulates and Protects TY1

**FIGURE 1:**
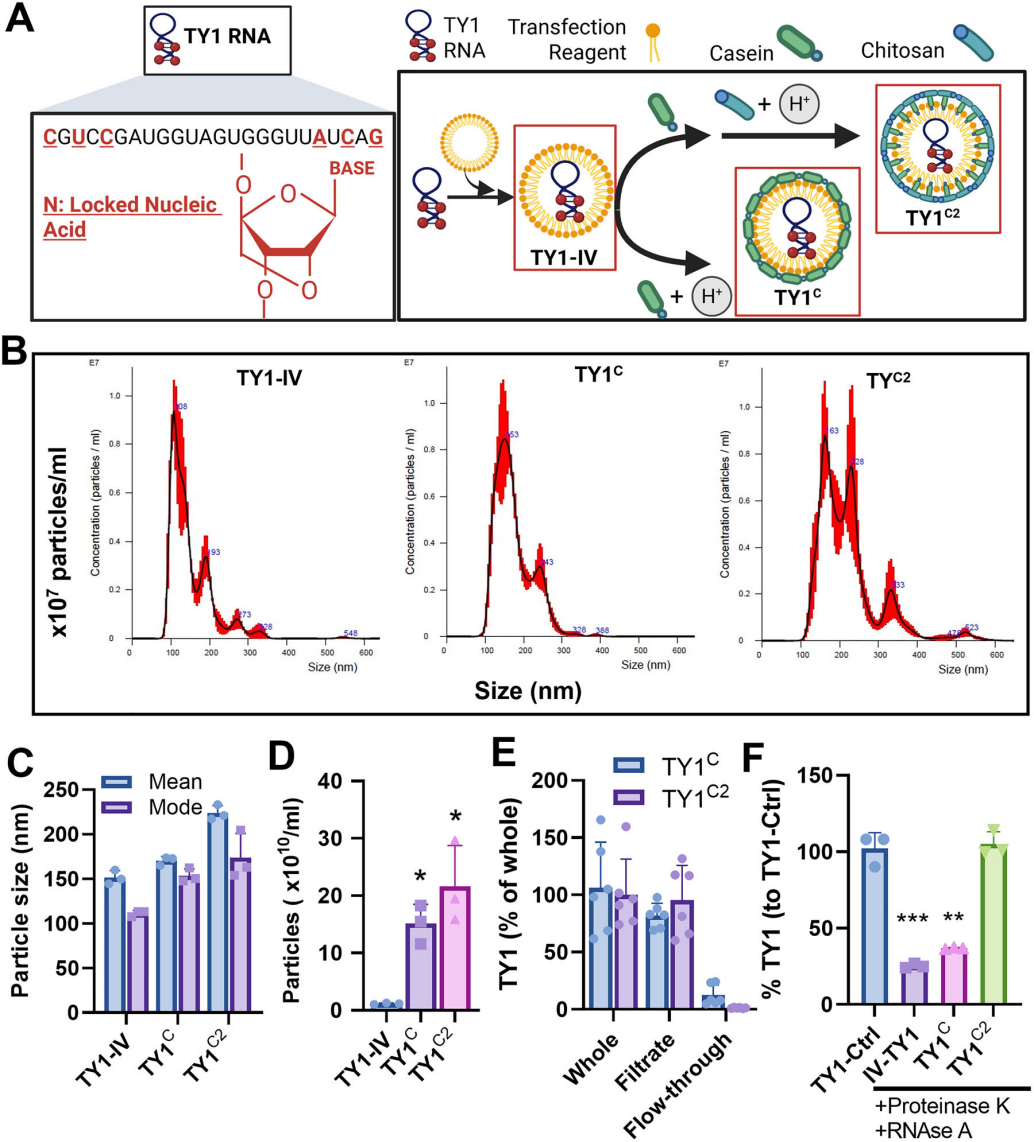
C2 Formulation of TY1. (A) Schematic for the formulation of TY1 in a lipid nanoparticle followed by encapsulation into casein and chitosan micelles. TY1‐IV comprises only the RNA and lipid nanoparticle (LNP), TY1^C^ comprises TY1‐IV formulated in casein, and TY1^C2^ comprises TY1‐IV formulated in casein and chitosan. (B–D) Quantification of size and concentration of TY1^C2^ micelles by dynamic light scattering (Nanoparticle tracking analysis; *n* = 3 biological replicates per group). (E) Assessment of the loading efficiency of TY1 in casein‐chitosan micelles. TY1^C2^ whole formulation was filtered using a 100 kD cutoff filter to separate encapsulated (filtrate) and free (flow‐through) TY1. Samples were then measured for TY1 abundance by qPCR (*n* = 3 biological replicates per group). (F) Enzyme degradation assay demonstrating resistance of TY1^C2^ to degradation by RNase A and Proteinase K (*n* = 3 biological replicates per group). Bars represent means and error bars represent s.d. Significance was determined by one‐way ANOVA; *p < 0.05; **p < 0.01; ***p < 0.001.

TY1 RNA, when packaged in LNP formed by DharmaFECT (henceforth TY1‐IV), is cardioprotective when administered intravenously post‐MI (Ibrahim et al. 2024). The oral formulation consists of two additional components: casein, the primary protein in milk, and chitosan, a naturally occurring polysaccharide. Casein arose from the finding that extracellular vesicles (EVs) encapsulated in casein were orally bioactive in a mouse model of Duchenne muscular dystrophy (Aminzadeh et al. 2021). We further added chitosan, given that the addition of a cationic polymer mediates the coagulation of casein micelles without changes in pH (Ausar et al. 2001; Narambuena et al. 2005). Casein/chitosan (C2) microparticle preparations efficiently encapsulate small molecular drugs and nucleic acids (Panão Costa et al. 2019) in aqueous solution (Ausar et al. 2001). Biopolymers including chitosan serve to enhance drug delivery across a variety of routes including oral, nasal, intravenous and ocular (Wang et al. 2011). The combination of casein and chitosan forms exceptionally stable micelles for biotherapeutic delivery (Jensen et al. 2020). Leveraging these observations, we first mixed TY1‐IV in a casein solution, then added medium molecular weight chitosan under acidic conditions to create TY1^C2^ (Figure 1A). Nanoparticle tracking analysis of TY1^C2^ particles demonstrated a mean diameter of ∼200 nm, which was slightly larger than TY1‐IV or TY1 formulated with casein only (TY1^C^; Figure 1B,C), with a higher particle concentration (Figure 1D). Consistent with previous reports (Ausar et al. 2001), encapsulation of the C2 formulation was highly efficient (95%; Figure 1E). To assess if the C2 formulation protects against enzymatic degradation, TY1‐IV, TY1^C^ or TY1^C2^ were mixed with RNase A and Proteinase K, and incubated for 20 min at 37°C. Only TY1^C2^ showed resistance to enzymatic degradation (Figure 1F). Thus, C2 formulation efficiently encapsulates and protects TY1.

### 2.2 Chronic Administration of TY1^C2^ Is Well Tolerated in Healthy Animals

**FIGURE 2:**
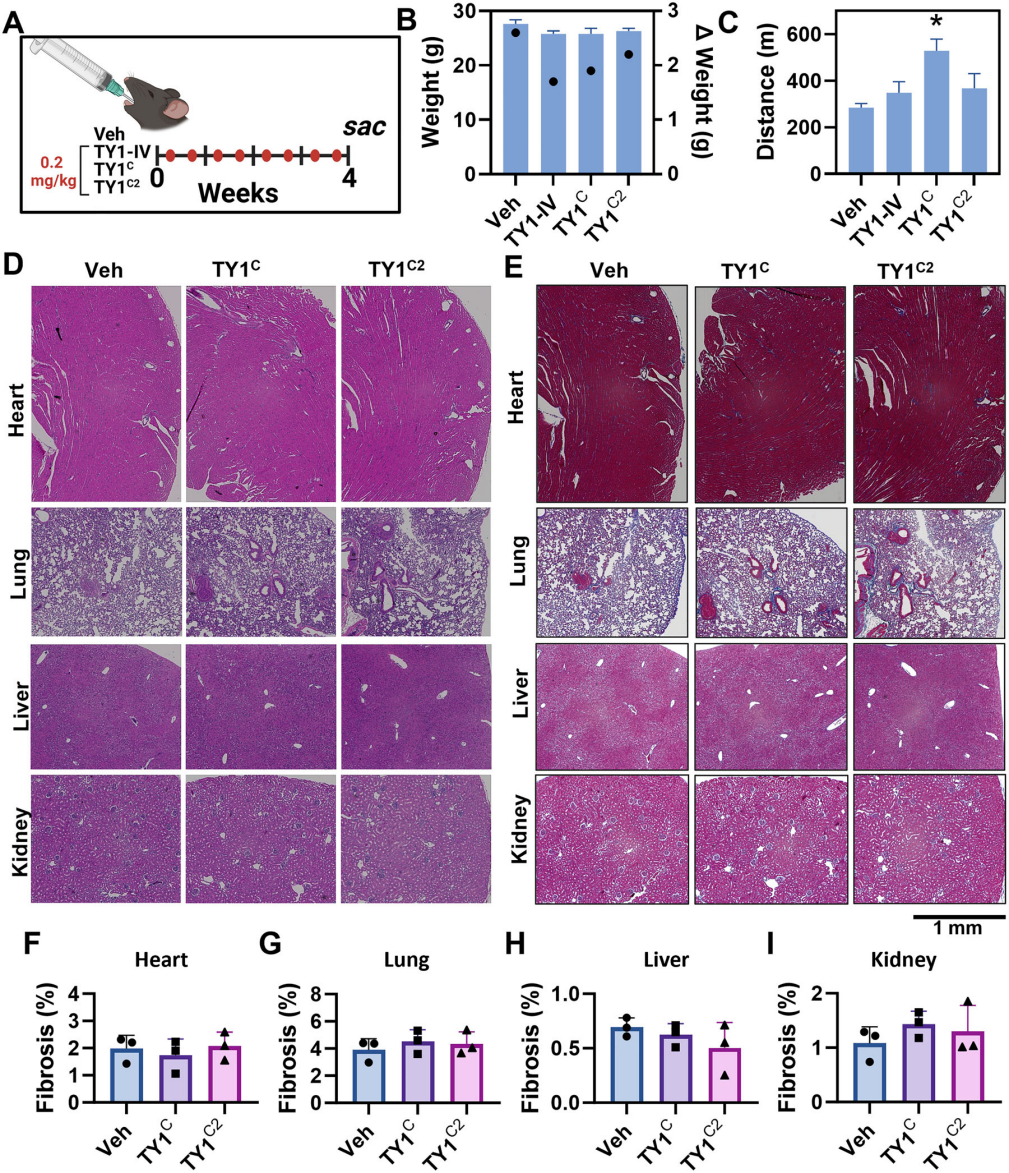
Long‐term oral administration of TY1^C2^ is well tolerated in healthy animals. (A) Schematic for assessing the toxicity of multiple administrations of variations of TY1 formulations in healthy animals (*n* = 5 animals per group). C57BL/6 mice were fed vehicle (PBS) or TY1^C2^ (0.2 mg/kg) twice a week for 4 weeks (eight total doses) followed by euthanasia. Other groups of animals were given TY1‐IV formulated in casein only (0.2 mg/kg; TY1^C^) or TY1 packaged in lipid nanoparticles (0.15 mg/Kg; TY1‐IV). (B) Average animal weights for each group (left axis: bars) and change in body weight (left axis: dots). (C) Exercise endurance as measured by a treadmill test. (D and E) Histological analysis of animal organs 4 weeks post‐exposure to assess inflammation (H & E; D) and fibrosis (Masson's trichrome; E, pooled data in F–I). Bars represent means and error bars represent s.d. Significance was determined by one‐way ANOVA; *p < 0.05.

To assess toxicity and tolerability, we gavaged healthy wild‐type mice with various formulations of TY1. These formulations contained various constituents of the C2 formulations to isolate any possible toxic effects of each excipient (transfection reagent, casein or chitosan). Thus we gavaged mice twice‐weekly with TY1‐IV (TY1 with DharmaFECT), TY1^C^ (TY1 with DharmaFECT and casein), or the full formulation, TY1^C2^ (TY1 with DharmaFECT, casein and chitosan) (Ibrahim et al. 2024). All groups were given 0.2 mg/kg, a dose close to the previously identified IV dose of 0.15 mg/kg (Ibrahim et al. 2024) but increased slightly to offset loss in loading efficiency in C2 micelles. After 4 weeks of exposure, animals were evaluated for exercise capacity, weighed and then euthanised for blood and tissue collection (Figure 2A). At endpoint, the groups were comparable in terms of weight (Figure 2B) and exercise capacity (Figure 2C; the enhanced exercise capacity in TY1^C^ mice is likely spurious, given the lack of therapeutic efficacy seen below with this formulation). Furthermore, no significant changes were seen in blood chemistry, lipid panel or liver enzymes (Table S1). Histological analyses of heart, lung, liver, kidney, and spleen revealed no increases in inflammatory infiltration or fibrosis (representative H&E [Figure 2D] and Masson's trichrome stained images [Figure 2E], and pooled data [Figure 2F–I]). Thus, none of the constituents of the TY1^C2^ formulation elicited toxic effects, and chronic TY1^C2^ administration is well‐tolerated in healthy animals.

### 2.3 TY1^C2^ Protects Heart and Lung Tissue Post‐Injury

**FIGURE 3:**
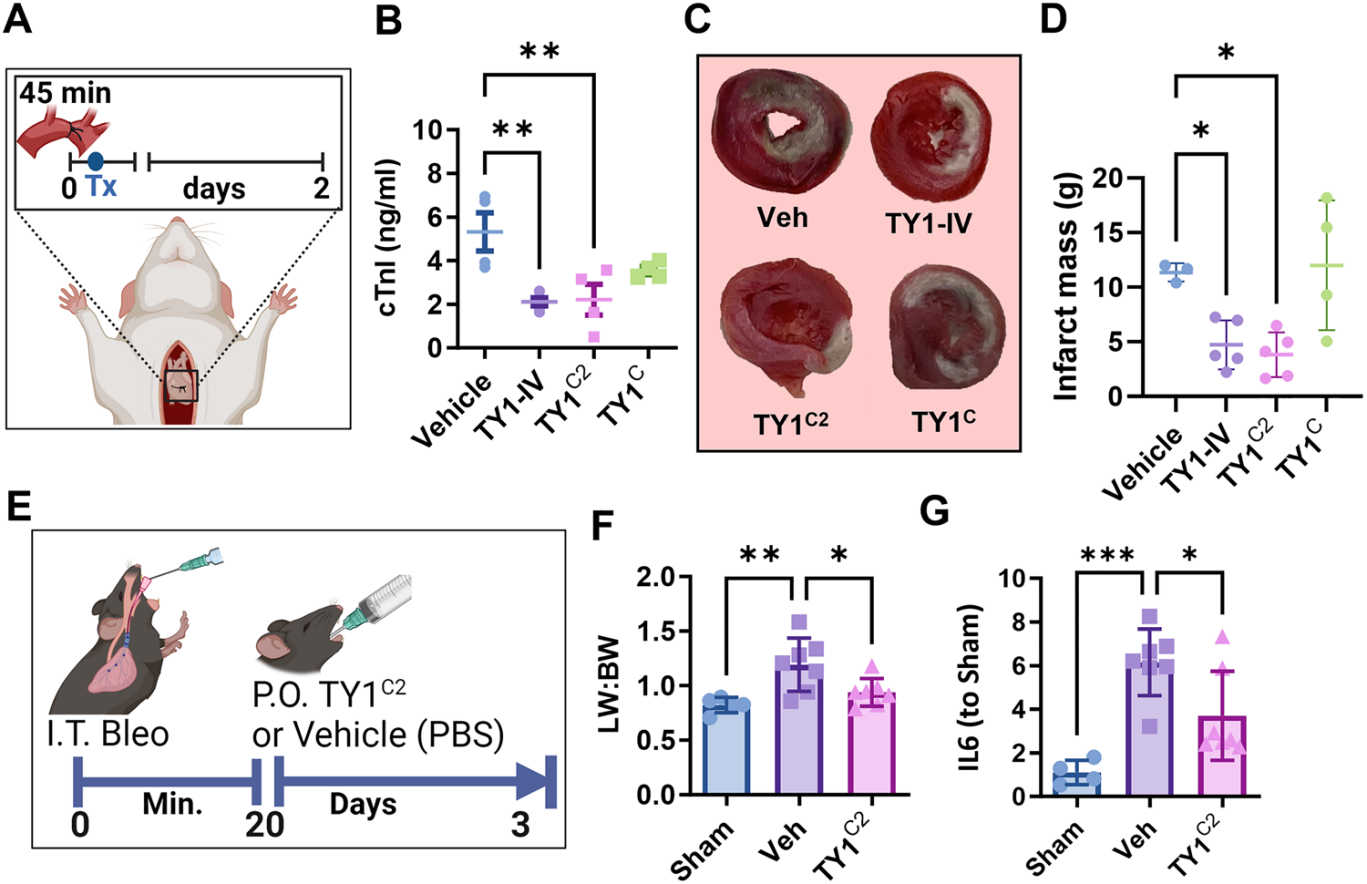
TY1^C2^ exerts disease‐modifying bioactivity in models of acute organ damage. (A) Schematic for the rat model of myocardial infarction. Rats were subjected to ligation of the left descending coronary artery for 45 min. Twenty minutes after reperfusion animals received vehicle (oral), TY1‐IV (intravenous), TY1^C2^ (oral) or TY1 formulated with LNPs (Dharmafect) and casein only (oral; TY1^C^, *n* = 4–5 rats per group). Forty‐eight hours post‐injury, animals fed TY1^C2^ had reduced cardiac tissue damage as demonstrated by reduced circulating levels of cardiac troponin as measured by ELISA (B) and infarct size as quantified by TTC staining (representative images; C and pooled data; D). (E) Schematic for the mouse model of bleomycin‐induced acute lung injury (*n* = 5–7 mice per group). Ten‐week‐old C57BL/6 mice received a single intratracheal administration of saline (sham) or bleomycin sulphate (0.7 mg/kg). Twenty minutes later, injured animals received oral vehicle (casein‐chitosan micelles only), or TY1^C2^ (0.2 mg/kg). Seventy‐two hours later, animals that had received a single oral dose of TY1^C2^ showed reduced lung inflammation as measured by lung weight to body weight ratio (F) and IL6 expression in lung tissue as measured by qPCR (G). Bars represent means and error bars represent s.d. Significance was determined by one‐way ANOVA; *p < 0.05; **p < 0.01; ***p < 0.001.

Intravenous TY1‐IV reduces myocardial damage in multiple models of MI (Ibrahim et al. 2024). To compare the efficacy of oral administration, a single oral dose of TY1^C2^ or vehicle (Veh^C2^, i.e., the C2 formulation without RNA) was delivered by gavage in a rat model of MI, 20 min after reperfusion (Figure 3A). Intravenous TY1‐IV, also administered 20 min after reperfusion, served as a positive control (Ibrahim et al. 2024). Animals that received TY1^C2^ orally showed reductions of the circulating ischemic biomarker cardiac troponin I (compared to Veh^C2^; Figure 3B) and infarct size (Figure 3C,D). Gavage with a casein‐only formulation (i.e., TY1^C^) yielded an inconsistent therapeutic benefit, unlike the robust efficacy of oral TY1^C2^, which, remarkably, is equivalent to that of intravenous TY1.

To determine if TY1^C2^ can exert broad disease‐modifying bioactivity, we investigated TY1^C2^ in a bleomycin‐induced acute lung injury model. Seven‐ to 10‐week‐old mice received a single intratracheal instillation of bleomycin sulphate followed by a single oral gavage of vehicle or TY1^C2^ 20 min later (Figure 3E). Lung tissue isolated from TY1^C2^ animals showed attenuated inflammation as measured by reduced lung weight to body weight ratio (Figure 3F) and attenuated expression of Il6, a pro‐inflammatory cytokine (Figure 3G).

### 2.4 Orally‐Delivered TY1^C2^ Is Taken Up by Macrophages in the Small Intestine and Accumulates in Peripheral Blood Mononuclear Cells

A qPCR‐based method is useful for tracking the biodistribution of EVs by amplifying NT4, a naturally occurring RNA with a sequence 96% identical to that of TY1 (Ciullo et al. 2022). We used the same method here to track the biodistribution of orally‐delivered TY1^C2^ in healthy animals. Despite the sensitivity of this technique (Ibrahim et al. 2024), TY1 was neither detectable in tissue nor in plasma (Figure S1A). TY1 accumulation remained modest even when the administered dose was increased 100‐fold, raising the possibility that orally administered TY1^C2^ is not absorbed into the circulation (Figure S1B). To investigate whether another orally‐administered RNA can enter the systemic circulation, we used an siRNA against coagulation factor VII (siFVII), which is produced by hepatocytes, the cell target for intravenously or intraperitoneally‐injected RNA (Ciullo et al. 2022). Twenty‐four hours after animals were injected intravenously or intraperitoneally with LNP‐packaged siFVII, liver tissue exhibited potent dose‐dependent suppression of Factor VII (Figure S1C,D). However, liver tissue from animals that had received oral siFVII formulated in C2 showed no suppression of Factor VII, even at doses much higher than those effective parenterally (Figure S1C,E). Similarly, an siRNA against a more ubiquitous gene target, Gapdh (siGap), worked well parenterally but not orally (Figure S1F,G). Thus, when given orally, two other small RNAs formulated in C2 had no systemic effects. All three compounds tested—TY1, siFVII and siGap—are undetectable in plasma when given orally in the C2 formulation, but oral TY1 is unique among these in exerting strong physiological effects in vivo, comparable to those of intravenous delivery. Thus, the C2 formulation does not provide oral access of its payload to circulating plasma, but something about TY1^C2^ nevertheless renders it orally bioavailable.

**FIGURE 4:**
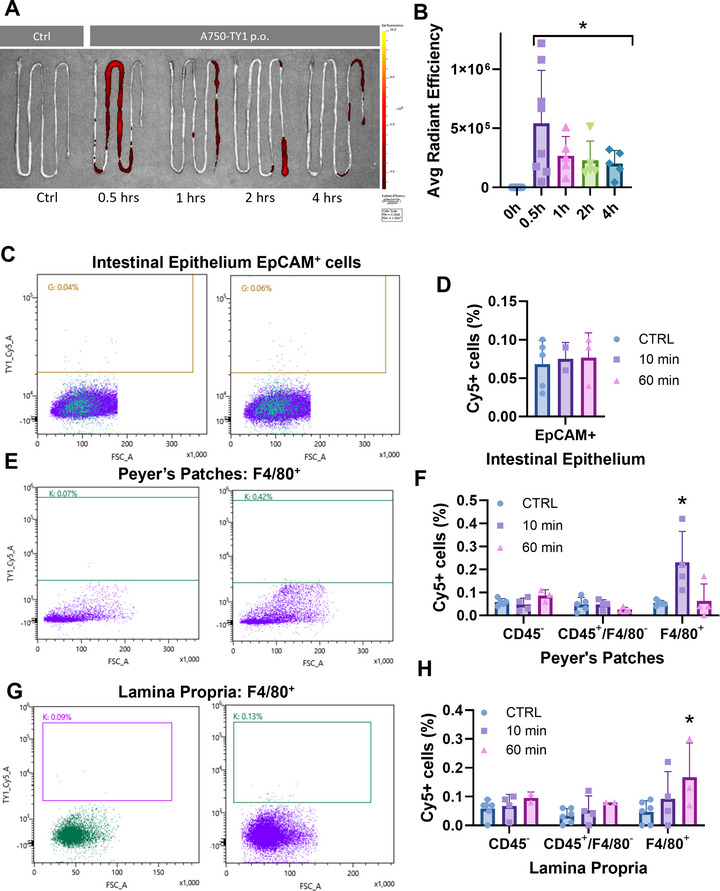
Intestinal macrophages take up TY1^C2^ after oral delivery. (A) Ex vivo imaging of small intestine from mice fed TY1^C2^ made with Alexa Fluor 750‐tagged TY1 (^A750^TY1^C2^) at 30‐, 60‐ and 120‐min post‐gavage. (B) Pooled data of TY1C2 signal (expressed as average radiant efficiency) in the small intestine over time (*n* = 5–8 mice per group). Flow cytometry analysis of ^Cy5^TY1^C2^ uptake by intestinal epithelial cells (EpCAM^+^; C and D), Peyer's patch cells (E and F), and lamina propria cells (G and H; *n* = 4 animals per group) at one hour post‐gavage. Bars represent means and error bars represent s.d. Significance was determined by one‐way ANOVA; *p < 0.05.

While siFVII and siGap block translation of their targeted genes in hepatocytes, TY1 is active in a different cell type: macrophages. TY1 is taken up by macrophages, which are necessary and sufficient to confer the therapeutic benefits of TY1 (Ibrahim et al. 2024). We therefore investigated the possibility that oral TY1^C2^ might be taken up by macrophages in the GI immune system, specifically those in the small intestine. Animals fed TY1^C2^ had unique enrichment of TY1 (as measured by qPCR) in Peyer's patches and, to a lesser extent, in nearby intestinal tissue (Figure S2A). Another, distant secondary lymphoid organ, the spleen, showed no TY1 (Figure S2A). This opened the possibility for visualising fluorescent‐labelled TY1^C2^. A pilot study evaluating signal intensity at 1 h post‐feeding (as informed by qPCR data in Figure S2A) demonstrated the feasibility of this approach (Figure S2B). Tracking fluorescent Alexa Fluor 750‐tagged TY1^C2^ (^A750^TY1^C2^), small intestine isolated from animals fed ^A750^TY1^C2^ showed a strong signal at 30 min, which waned by 4 h (Figure 4A,B). Gut‐associated lymphoid structures, including Peyer's patches, are inductive sites where intestinal contents interface with the immune system (Kunisawa et al. 2012). Among cells isolated from the small intestine 1 h post‐gavage, intestinal epithelial cells showed no TY1 uptake (EpCAM; Figures S3A and 4C,D). In contrast, macrophages (F4/80^+^) exhibited robust TY1 signal in both Peyer's patches (Figures S3B and 4E,F) and in the lamina propria (Figures S3C and 4G,H). Oral administration of Cy5‐conjugated TY1 (without C2 formulation) showed no uptake by intestinal tissue (Figure S3D). We also looked for evidence of TY1 in peripheral blood mononuclear cells (PBMCs) after oral administration of TY1^C2^. PBMCs traffic through intestinal tissue and crosstalk with resident intestinal macrophages (Delfini et al. 2022). As observed previously, TY1 was not detected in any organ tissue at any time point after oral administration (Figure S4A). Nevertheless, TY1 was detected in PBMCs starting 3 h after an oral dose, and persisted for up to 72 h (Figure S4B). Fractionation of PBMCs further revealed uptake of TY1 by CD11b+ cells (monocytes). The fact that monocytes are macrophage precursors rationalises previous observations that macrophages are necessary and sufficient to mediate the therapeutic effects of TY1 (Figure S4C). As consistency check, we looked for downstream targets of TY1 signalling including pro‐inflammatory cytokine expression (Ibrahim et al. 2024). We isolated monocytes from animals fed TY1 or control (C2 formulation only; Figure S4D). At 24 h post‐oral administration monocytes and macrophages isolated from animals fed TY1 had significantly lower levels of pro‐inflammatory targets including IL1α, TNFα and IFNγ (Figure S4D,E). Thus, orally administered TY1^C2^ is taken up by macrophages in the small intestine. Somehow, by as‐yet‐undetermined mechanisms, this localised GI lymphoid uptake leads to uptake by PBMCs and subsequent reparative benefits in distant tissue, as seen in MI and acute lung injury (Figure 3). Similarly broad‐ranging therapeutic benefits have been described after oral delivery of TY1^C2^ in mice with heart failure and preserved ejection fraction (Miyamoto et al. 2024).

## 3 Methods

### 3.1 Nanoparticle Tracking Analysis

The size and concentration of TY1^C2^ nanoparticles were characterised using nanoparticle tracking analysis by NanoSight NS300 (Malvern Panalytical, Malvern, UK).

### 3.2 Experimental Animals

All studies were performed at Cedars‐Sinai Medical Center in accordance with the Institutional Animal Care and Use Committee guidelines. Six to 10‐week‐old male or female C57BL/6J mice (Jackson Laboratory, Maine, USA) were used for safety evaluation, biodistribution study, and bleomycin‐induced lung injury model. Seven to 10‐week‐old Wistar‐Kyoto female rats (Envigo, Indiana, USA) were used for the MI model.

### 3.3 TY1 Intravenous (TY1‐IV) Injection

Animals were treated with TY1 oligo ribonucleotide (0.15 mg/kg body weight) after adding DharmaFECT1 (0.36 mL/kg body weight, T‐2001, Horizon Discovery) by retro‐orbital injection. Injections were performed on alternating eyes (no more than 3 injections per eye) under general anaesthesia; no signs of ocular injury were observed.

### 3.4 TY1 Oral Formulation and Gavage

Each animal was fed (by gavage) TY1‐IV (TY1 in lipid nanoparticles [Dharmafect]) formulated in casein‐chitosan (C2). Briefly, TY1‐IV (0.2 mg/kg body weight) was mixed with a 5% casein solution from bovine milk (125 µL per mouse, C4765, Millipore Sigma) and incubated for 15 min at room temperature with gentle agitation. Following incubation, 100 µL of a 0.1% acetic acid solution (695092, Sigma‐Aldrich)/0.2% chitosan (448869, Millipore Sigma) was added dropwise and mixed well. After 60 min incubation at room temperature, the solution was administered via an oral gavage needle (01‐290‐3B, Fisher Scientific); the animal was held with the body tilted upward, and the needle was inserted into the mouth gently.

### 3.5 Enzyme Degradation Assay

TY1‐IV, TY1^C^ and TY1^C2^ were mixed with 5 µg/mL of RNase A (19101, QIAGEN) and 1 mg/mL of Proteinase K (EO0491, Fisher Scientific), and incubated for 20 min at 37°C using a tube rotator to gently mix samples during incubation. As a control, TY1‐IV without RNase A and Proteinase K was incubated in the same manner. TY1 was purified from samples using miRNeasy Mini Kit (217004, QIAGEN) according to the manufacturer's protocol with minor modifications. Briefly, samples were lysed and homogenised by adding 700 µL of QIAzol and vortexing for 30 s. Spike‐In Control (5.6 × 10^8^ copies, 219610, QIAGEN) was added to the samples for assessment of recovery of small RNAs during the process. Samples were then mixed with 140 µL of chloroform and centrifuged at 12,000 × *g* for 15 min at 4°C. After centrifugation, the upper aqueous phase was mixed with 1.5 volumes of 100% ethanol and transferred to an RNeasy Mini spin column. Washing steps using Buffer RWT/RPE were performed according to the manufacturer's protocol. A total of 40 µL of RNase‐free water was added to the column membrane and the column was centrifuged at 8000 × *g* for 1 min to elute the RNA. RT‐qPCR was performed as described below.

### 3.6 Toxicity Study

Safety evaluation was conducted by administrating TY1^C2^ to healthy mice orally twice a week for 4 weeks. Exercise endurance (details below) and body weight were measured at the endpoint followed by isolation of organ samples. Harvested tissues were embedded in paraffin and cut into sections, which were stained with haematoxylin and eosin or Masson's trichrome. Blood chemistry tests were performed by Antech (California, USA).

### 3.7 Rat Model of Myocardial Infarction

All rats were housed in a pathogen‐free facility (cage bedding: Sani‐Chips, PJ Murphy) with a 14/10 h light/dark cycle with food (PicoLab Rodent Diet 20 [no. 5053], LabDiet) and water provided ad libitum. In vivo, experimental protocols were performed on 7 to 10‐week‐old female Wistar‐Kyoto rats. To induce MI, a thoracotomy was performed at the fourth intercostal space to expose the heart under general anaesthesia. A 7–0 silk suture was then used to ligate the left anterior descending coronary artery, which was removed after 45 min to allow for reperfusion. Twenty minutes later, animals received vehicle (PBS only) or TY1^C2^ (0.2 mg/kg as described previously) or TY1 formulated only in casein (TY1^C^; 0.2 mg/kg). As a positive control, a group of animals received TY1‐IV (0.15 mg/kg) injected in the retro‐bulbar space.

### 3.8 TTC Staining

Two days post‐MI, 10% KCl was injected into the left ventricle to arrest hearts in diastole. Then, hearts were harvested, washed in PBS, and cut into 1‐mm sections from apex to base, above the infarct zone. Sections were incubated with 1% 2,3,5‐triphenyl‐2H‐tetrazolium chloride solution (TTC, Sigma‐Aldrich) for 30 min at 37°C in the dark and washed with PBS. Then, sections were imaged and weighed. The infarcted zones (white) were delineated from viable tissue (red) and analysed (ImageJ software). Infarct mass was calculated in the tissue sections according to the following formula: (infarct area/total area)/weight (mg).

### 3.9 ELISA

Blood was collected from animals at the study endpoint in EDTA tubes. After being left undisturbed at 4°C for 30 min, plasma was obtained after 15‐min centrifugation at 4000 rpm. Cardiac TnI was quantified using the RAT cardiac troponin‐I ELISA kit (cTNI‐2‐HSP, Life Diagnostics) according to the manufacturer's protocol. IL‐6 and BNP plasma levels were analysed using the following ELISA kit: Mouse IL‐6 Quantikine ELISA Kit (M6000B, R&D systems), Mouse BNP EIA (EIAM‐BNP‐1, RayBiotech) according to manufacturers’ instructions.

### 3.10 Mouse Model of Bleomycin‐Induced Lung Injury

Ten‐week‐old female C57BL/6 mice were given a single intratracheal instillation of a 50 µL solution of bleomycin sulphate (0.7 mg/kg) under general anaesthesia. Twenty minutes later, animals were orally administered 230 µL of vehicle (casein‐chitosan‐LNP micelles formulated without TY1) or TY1^C2^. At 72 h post‐injury, animals were weighed and sacrificed, and lungs were harvested for weighing and RNA isolation for subsequent qPCR to measure Il6 expression.

### 3.11 Exercise Endurance (treadmill) Test

Mice were placed on an Exer 3/6 rodent treadmill (Columbus Instruments, Ohio, USA) at a 5‐degree elevation. At first, the speed was increased by 5 m/min for 2 min, and the speed remained 10 m/min for 5 min. Then the speed was increased by 2 m/2 min until mice were exhausted, as defined as the inability of the mouse to return to the treadmill from the shock grid for 10 s.

### 3.12 RNA Isolation and RT‐qPCR

Total RNA was extracted from mouse tissue using RNeasy plus kit (74136, QIAGEN) and Maxtract High‐density tubes (129056, QIAGEN). cDNA was synthesised from RNA using a High‐capacity cDNA reverse transcription kit (4368813, Applied Biosystems) according to the manufacturer's protocol. Real‐time PCR (QuantStudio 12K Flex Real‐Time PCR system; Thermo Fisher Scientific) was performed in triplicate using the following TaqMan Gene Expression Assay probes: IL‐6 (Mm00446190_m1) and B2m (Mm00437762_m1) analyzed by the ddCt method.

### 3.13 TY1 Quantification by qPCR

TY1 was extracted from mouse tissue using miRNeasy mini kit (217004, QIAGEN) or miRNeasy Serum/Plasma kit (217184, QIAGEN) according to the manufacturer's protocol. cDNA was synthesised from RNA using miRCURY LNA RT Kit (339340, QIAGEN) according to the manufacturer's protocol. All RNA samples were normalised using a spike RNA control (UniSp6: GeneGlobe ID: YP00203954). Real‐time PCR was performed in duplicate or triplicate using the following kits and primers; miRCURY SYBR Green PCR Kit (339345, QIAGEN), miRCURY LNA miRNA PCR Assay (339306, QIAGEN), miRCURY LNA miRNA Custom PCR Assay (339317, QIAGEN).

### 3.14 Oral‐siRNA Bioavailability Study

Ambion in vivo siRNA targeting FVII (4459408, Fisher Scientific), Gapdh (4459407, Thermo Fisher Scientific), or vehicles were injected intravenously or intraperitoneally (0.1–2 mg/kg body weight) according to the manufacturer's protocol. For oral administration, siRNAs were mixed with transfection reagent (Invivofectamine 3.0, IVF3001, Thermo Fisher Scientific) in a 1.2:1 (µg:µL) ratio followed by incubation for 30 min at 50°C. C2‐formulated siRNAs were then made by adding 5% casein solution and 0.2% chitosan/1% acetic acid solution as previously described (cf. Figure 1A). Organs including the liver were harvested 24 or 72 h after treatment with siRNAs. Livers were digested using Bead Mill 4 (Fisher Scientific), and RNA purification and cDNA synthesis were performed as described above. qPCR was performed using the following Taqman Gene Expression Assay (Thermo Fisher Scientific): Coagulation factor VII (F7) (Mm00487329_m1) and Glyceraldehyde 3‐phosphate dehydrogenase (Gapdh) (Mm99999915_g1).

### 3.15 In Vivo Imaging System (IVIS)

Small intestinal tissues were harvested from mice 30 min, 1 h, 2 h, and 4 h after treatment with C2‐formulated TY1 conjugated with Alexa Fluor 750. The lumen of the intestine was thoroughly flushed with PBS using a 10 mL syringe and 14‐gauge catheter to get rid of wastes and any residuals inside. Intestinal tissues were then cut through longitudinally and any visible residuals at the inner lining were further removed. Tissues were then rigorously rinsed with PBS four times and stored on ice for optical imaging. A radiant efficiency [photons/sec/cm^2^/str)/(µW/cm^2^]) of the fluorescence from the tissues was measured with an IVIS Lumina XR (Revvity, Massachusetts, USA) using 745/800 nm emission filter set with a fixed exposure time. Five to 8 samples were collected per group for quantitative analysis.

### 3.16 Flow Cytometry

Small intestines were harvested from mice 1 h oral gavage with C2‐formulated TY1 conjugated with Cy5 or vehicle and rinsed with PBS as described above. Cells were extracted from intestinal tissues using the Lamina Propria Dissociation Kit (130‐097‐410, Miltenyi) to make a single‐cell suspension according to the manufacturer's protocol with minor modifications. Briefly, tissues were cut laterally into pieces of ∼0.5 cm in length and incubated with predigestion solution for 20 min at 37°C. Tissues were then vortexed and applied onto a 100 µm cell strainer. Flow through was kept on ice for the collection of epithelial cells. Tissues were further incubated with HBSS for another 20 min and flow‐through after filtration with a cell strainer was kept on ice. Tissues were then incubated with enzyme mix in digestion solution for 30 min at 37°C. After rigorous vortexing and pipetting, the cell suspension was applied onto a 100 µm cell strainer, and flow through was centrifuged at 300 × *g* for 5 min at room temperature. The cells were resuspended in 1 mL of ACK Lysing Buffer and incubated for 1 min at room temperature to remove erythrocytes. Cells were then pelleted and resuspended with PBS. 2 × 10^6^ cells were isolated for staining. Cells were incubated with 1 µL of Ghost Dye Red 710 (13‐0871, Cytek Biosciences) for 30 min at 4°C. Cells were pelleted and resuspended in 100 µL of Cell Staining Buffer (420201, BioLegend) followed by incubation with 1 µL of TruStain FcX PLUS (156603, BioLegend) for 15 min at 4°C to block non‐specific Fc‐mediated interactions. Cell suspensions were mixed with antibodies (1 µL of FITC‐CD45 (11‐0541‐82, Thermo Fisher Scientific), 1.25 µL of PE‐F4/80 (12‐4801‐82, Thermo Fisher Scientific), 5 µL of Alexa 594‐EpCAM (BS‐4889R‐A594, Thermo Fisher Scientific) and 10 µL of APC‐Microfold (130‐102‐149, Miltenyi Biotec)) and incubated for 30 min at 4°C. Cells were washed two times by resuspension in 2 mL of Cell Staining buffer and centrifugation at 800 × *g* for 5 min.  Finally, cells were resuspended in 500 µL of FluoroFix Buffer (422101, BioLegend). Unstained and isotype control samples were also prepared.

### 3.17 Statistical Analysis

Statistical parameters including the number of samples (*n*), descriptive statistics (mean and standard deviation), and significance are reported in the figures and figure legends. In general, at least *n* = 3 was used for each time point and each experiment. Differences between groups were examined for statistical significance using Student's *t*‐test or one‐way analysis of variance (ANOVA). Differences with *p* values < 0.05 were regarded as significant.

## 4 Discussion

RNA drug delivery is fraught with challenges. Efforts to overcome these challenges include extracellular vesicles which have shown promise in providing a protective carrier for small RNAs, native tissue targeting and biocompatibility (Kumar et al. 2024). RNA delivery through the oral route presents added challenges. RNA molecules and the lipid nanoparticles that encapsulate them can be degraded by acid in the stomach and nucleases in the gastrointestinal tract. Furthermore, RNA molecules, owing to their size and charge, are not able to permeate the intestinal epithelium, limiting bioavailability (Damase et al. 2021). Other strategies focus on chemical modifications of RNA and nanoparticle carriers to enhance their stability. Improvements in RNA include ribose modifications and cholesterol conjugates, while various nanoparticle excipients have been explored, with limited success (O'Driscoll et al. 2019). Regardless of chemical modifications, unprotected RNA molecules are largely degraded as they pass through the GI tract. Moreover, even if nanoparticles provide sufficient protection of RNA molecules, mucous penetration and subsequent absorption through the tight gap junctions of intestinal villi, remain as barriers (Yu et al. 2020).

Here, we demonstrate a polymer‐based formulation of a small non‐coding RNA with disease‐modifying bioactivity, TY1, for oral delivery. We found the C2 formulation to efficiently encapsulate TY1 and protect the oligonucleotide from the harsh GI environment. TY1^C2^ is well‐tolerated in healthy animals after chronic exposure. In two models of acute organ injury, a single oral administration of TY1^C2^ demonstrated tissue‐protective activity equivalent to that of intravenous TY1. Although C2 formulation does not deliver free RNA cargo to the systemic circulation, as gauged by plasma concentration, we do observe TY1 in circulating monocytes 3–72 h post‐ingestion. Thus, orally‐delivered TY1 is first taken up and sequestered by macrophages in the lamina propria and Peyer's patches; afterwards, the drug can be found in circulating monocytes (see **Graphical Abstract**).

Given the importance of macrophages—they are both necessary and sufficient for TY1 efficacy (Ibrahim et al. 2024)—it stands to reason that the intestinal macrophages that take up TY1^C2^ initiate the systemic therapeutic effect. A subsequent step, and one that helps rationalise the systemic benefits, is the appearance of TY1 in circulating monocytes. Precisely how TY1 is transferred from the GI immune system to circulating monocytes remains to be determined. A recent study of another orally delivered RNA proposed a relay system involving, in sequence, intestinal macrophages in the Peyer's patches, lymphocytes, dendritic cells and systemic circulation (Kim et al. 2023), but direct experimental evidence was lacking. Other studies suggest that macrophages in the intestine, once activated by immunostimulatory nanoparticles like yeast‐derived glucans, stimulate uptake of these nanoparticles by macrophages (Soto et al. 2012). Efficient oral delivery of siRNA targeting Map4K4 to intestinal macrophages has been reported to suppress systemic inflammation in a model of LPS‐induced sepsis; macrophages with suppressed Map4k4 were detected in distant tissues, including lung, spleen, muscle, liver and the peritoneum (Aouadi et al. 2009). Similarly, chitosan has been shown to exert immunostimulatory properties (Moran et al. 2018; Gong et al. 2022) and systemically deliver orally‐administered‐siRNA (Ballarín‐González et al. 2013). However, challenges with using chitosan exclusively for oral formulation include limited permeability across the intestinal epithelium (Sangnim et al. 2023). Conversely, casein facilitates uptake by cells and enhances sustained bioavailability (Elzoghby et al. 2011). Thus, when combined, the immunostimulatory properties of chitosan and bioavailability‐enhancing properties of casein underlie the C2 formulation's exceptional ability to deliver small RNA cargo to intestinal macrophages with effects that are, dose‐for‐dose, comparable to those of IV administration. The C2 formulation may, more broadly, enable the targeting of macrophages as a therapeutic strategy, not only for TY1 but also for other drugs that act on macrophages.

## Author Contributions

**Shukuro Yamaguchi**: investigation, writing – review and editing, methodology, formal analysis, validation, writing – original draft, data curation. **Kazutaka Miyamoto**: investigation, methodology, validation, formal analysis. **Xaviar M. Jones**: investigation. **Alessandra Ciullo**: investigation, methodology, formal analysis. **Kara Tsi**: investigation, methodology, formal analysis. **Jessica Anderson**: investigation, methodology, formal analysis. **Hiroaki Komuro**: investigation. **Salwa Soussi**: investigation. **Ashley Morris**: investigation, methodology, formal analysis. **Diana Kitka**: investigation. **De‐Zhao Liu**: investigation. **Anh Nguyen**: investigation. **Eduardo Marbán**: conceptualization, writing – original draft, writing – review and editing, supervision, resources. **Ahmed G. E. Ibrahim**: conceptualization, writing – original draft, supervision, resources, project administration, data curation.

## Conflicts of Interest

Eduardo Marbán owns founder's equity and Ahmed Ibrahim owns stock in Capricor Therapeutics which has no licensing rights to TY1 or related discoveries. All other authors declare no competing interests.

## Supporting information




**Supplementary Table 1: Blood chemistries from animals dedicated to TY1 toxicity study**. Metabolic panel of plasma samples from healthy animals that had been given vehicle, oral TY1 packaged in LNP only, TY1^C^, or TY1^C2^ twice a week for four weeks (n = 5 animals per group). Statistical Analysis was done using a One‐Way ANOVA with Tukey's post test to compare for multiple comparisons.
**Supplementary Figure 1: C2 formulation does not deliver small RNA cargo systemically**. (**A**) qPCR demonstrated a lack of TY1 in organ tissues at 0, 20, 60, and 100 minutes post oral administration of TY1^C2^ (0.2 mg/kg). (**B**) TY1 was still undetectable even when the oral dose was increased 100‐fold (20 mg/kg). (**C**) Schematic for delivering intravenous and orally formulated siRNA to assess effects on liver tissue. (**D**) Successful suppression of Factor VII in liver tissue following intravenous or intraperitoneal administration of siRNA against Factor VII (siFVII). (**E**) Oral administration of C2‐formulated siFVII at higher doses of siFVII failed to suppress Factor VII in the liver. (**F**) Intravenous administration of siRNA against Gapdh (siGap) led to successful suppression of Gapdh in liver tissue. (**G**) Oral administration of C2‐formulated siGap failed to suppress Gapdh in both liver tissue and resident liver macrophages. Bars represent group means and error bars represent s.d.
**Supplementary Figure 2: TY1^C2^ biodistribution in mouse tissue**. (**A**) qPCR of TY1 in Peyer's patches, intestinal tissue, and spleen demonstrating absorption of TY1^C2^ by Peyer's patches and (to a lesser extent) intestinal tissue (n = 4 animals per group). (**B**) Detectable fluorescence signal of ^A750^TY1^C2^ in the mouse small intestine one hour post oral delivery with notable absence in other organs.
**Supplementary Figure 3: TY1^C2^ uptake by intestinal macrophages (A)** Gating strategy for assessing uptake of ^Cy5^TY1^C2^ in intestinal epithelial cells (**B**), Peyer's patches (**C**), and Lamina propria. (**D**) Animals fed Cy5‐labelled TY1 show impaired uptake by intestinal tissue 60 minutes post‐oral gavage (n = 3 animals per group).
**Supplementary Figure 4: TY1 is abundant in PBMCs post oral administration of TY1^C2^**. (**A**) Lack of systemic accumulation of TY1 as measured by qPCR for TY1 in organ tissue (n = 5 animals per timepoint per group). (**B**) Time‐dependent accumulation of TY1 in peripheral blood mononuclear cells (PBMCs; n = 6 animals per group for all treatments, n = 3 animals for vehicle) and fractionated CD11b^+^ monocytes (n = 3‐5 animals per group; **C**) in animals given a single oral administration of TY1^C2^). (**D**) Schematic for isolating monocytes from animals fed TY1‐C2. (**E**) Gene expression analysis showing significant reduction of pro‐inflammatory cytokines including IL1β and TNFα, and trend towards reduced IFNγ (n = three animals per group). Bars represent group means and error bars represent SEM. Statistical analysis in E was done Student's t test with 95% CI. *p<0.05, **p<0.01, ***p<0.001.

## Data Availability

The data that support the findings of this study are available from the corresponding author upon reasonable request.
