# Supplementary material for: Oral Bioavailability of a Noncoding RNA Drug, TY1, That Acts on Macrophages
Source: J Extracell Biol. 2025 Aug 14;4(8):e70081. doi: 10.1002/jex2.70081 (PMC12353012; doi:10.1002/jex2.70081)

**Supplementary Material**

**Supplementary Figure Legends**

**Supplementary Table 1: Blood chemistries from animals dedicated to TY1 toxicity study.**

Metabolic panel of plasma samples from healthy animals that had been given vehicle, oral TY1 packaged in LNP only, TY1^C^, or TY1^C2^ twice a week for four weeks (n= 5 animals per group). Statistical Analysis was done using a One-Way ANOVA with Tukey’s post test to compare for multiple comparisons.

**Supplementary Figure 1: C2 formulation does not deliver small RNA cargo systemically.**

(**A**) qPCR demonstrated a lack of TY1 in organ tissues at 0, 20, 60, and 100 minutes post oral administration of TY1^C2^ (0.2 mg/kg). (**B**) TY1 was still undetectable even when the oral dose was increased 100-fold (20 mg/kg). (**C**) Schematic for delivering intravenous and orally formulated siRNA to assess effects on liver tissue. (**D**) Successful suppression of Factor VII in liver tissue following intravenous or intraperitoneal administration of siRNA against Factor VII (siFVII). (**E**) Oral administration of C2-formulated siFVII at higher doses of siFVII failed to suppress Factor VII in the liver. (**F**) Intravenous administration of siRNA against Gapdh (siGap) led to successful suppression of Gapdh in liver tissue. (**G**) Oral administration of C2-formulated siGap failed to suppress Gapdh in both liver tissue and resident liver macrophages. Bars represent group means and error bars represent s.d.

**Supplementary Figure 2: TY1^C2^ biodistribution in mouse tissue.**

(**A**) qPCR of TY1 in Peyer’s patches, intestinal tissue, and spleen demonstrating absorption of TY1^C2^ by Peyer’s patches and (to a lesser extent) intestinal tissue (n=4 animals per group). (**B**) Detectable fluorescence signal of ^A750^TY1^C2^ in the mouse small intestine one hour post oral delivery with notable absence in other organs.

**Supplementary Figure 3: TY1^C2^ uptake by intestinal macrophages (A)** Gating strategy for assessing uptake of ^Cy5^TY1^C2^ in intestinal epithelial cells (**B**), Peyer’s patches (**C**), and Lamina propria. (**D**) Animals fed Cy5-labelled TY1 show impaired uptake by intestinal tissue 60 minutes post-oral gavage (n=3 animals per group).

**Supplementary Figure 4: TY1 is abundant in PBMCs post oral administration of TY1^C2^**

(**A**) Lack of systemic accumulation of TY1 as measured by qPCR for TY1 in organ tissue (n=5 animals per timepoint per group). (**B**) Time-dependent accumulation of TY1 in peripheral blood mononuclear cells (PBMCs; n=6 animals per group for all treatments, n=3 animals for vehicle) and fractionated CD11b^+^ monocytes (n=3-5 animals per group; **C**) in animals given a single oral administration of TY1^C2^). (**D**) Schematic for isolating monocytes from animals fed TY1-C2. (**E**) Gene expression analysis showing significant reduction of pro-inflammatory cytokines including IL1β and TNFα, and trend towards reduced IFNγ (n=three animals per group). Bars represent group means and error bars represent SEM. Statistical analysis in E was done Student’s t test with 95% CI. *p<0.05, **p<0.01, ***p<0.001.


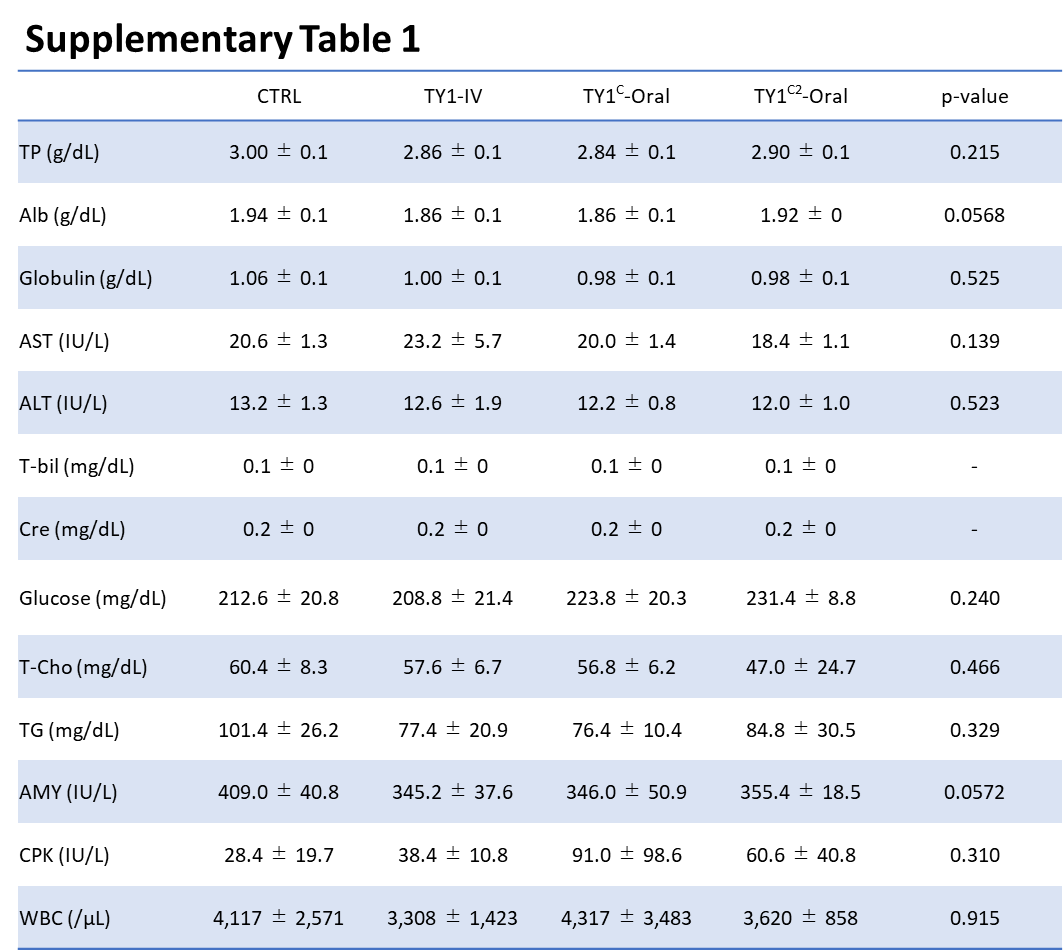


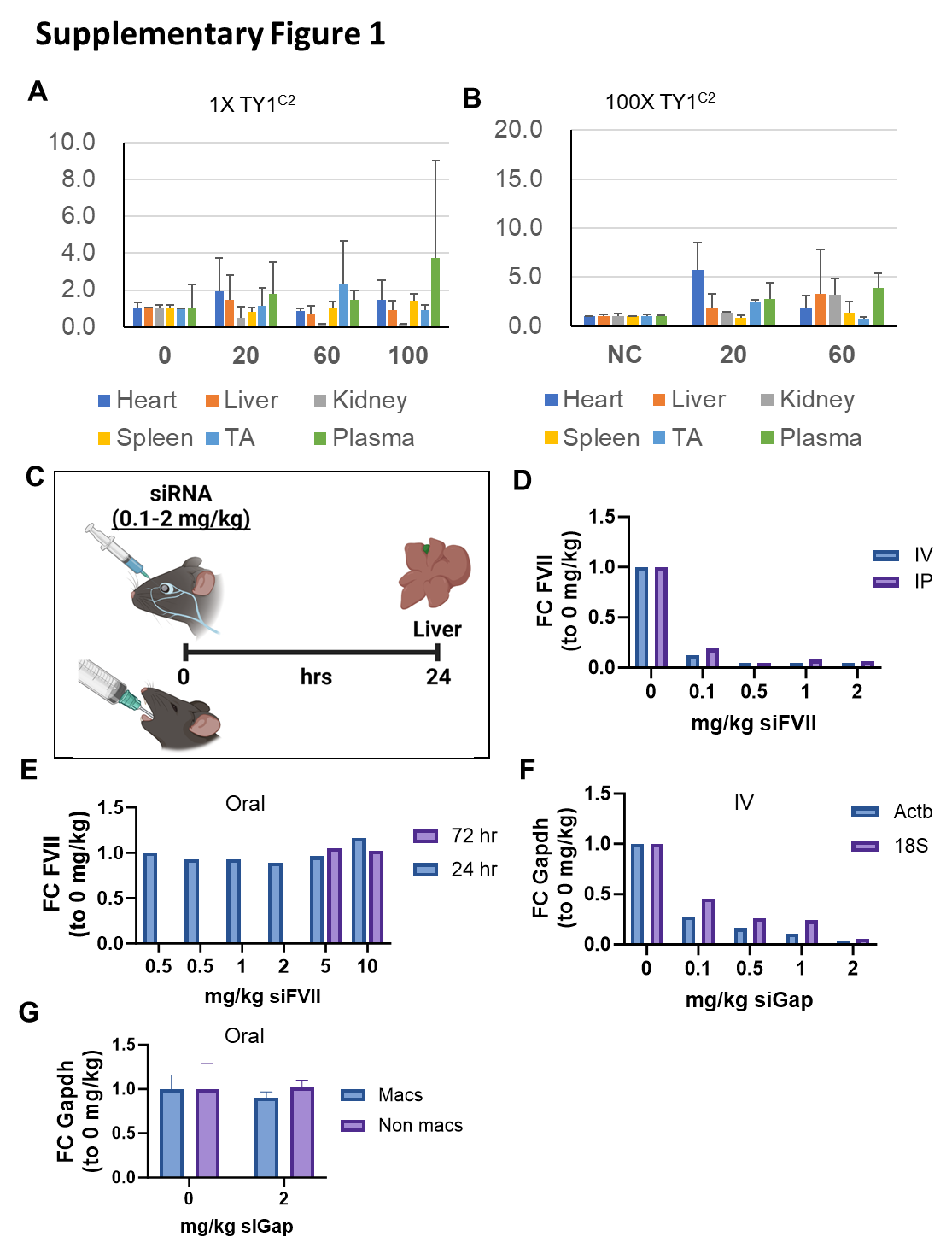


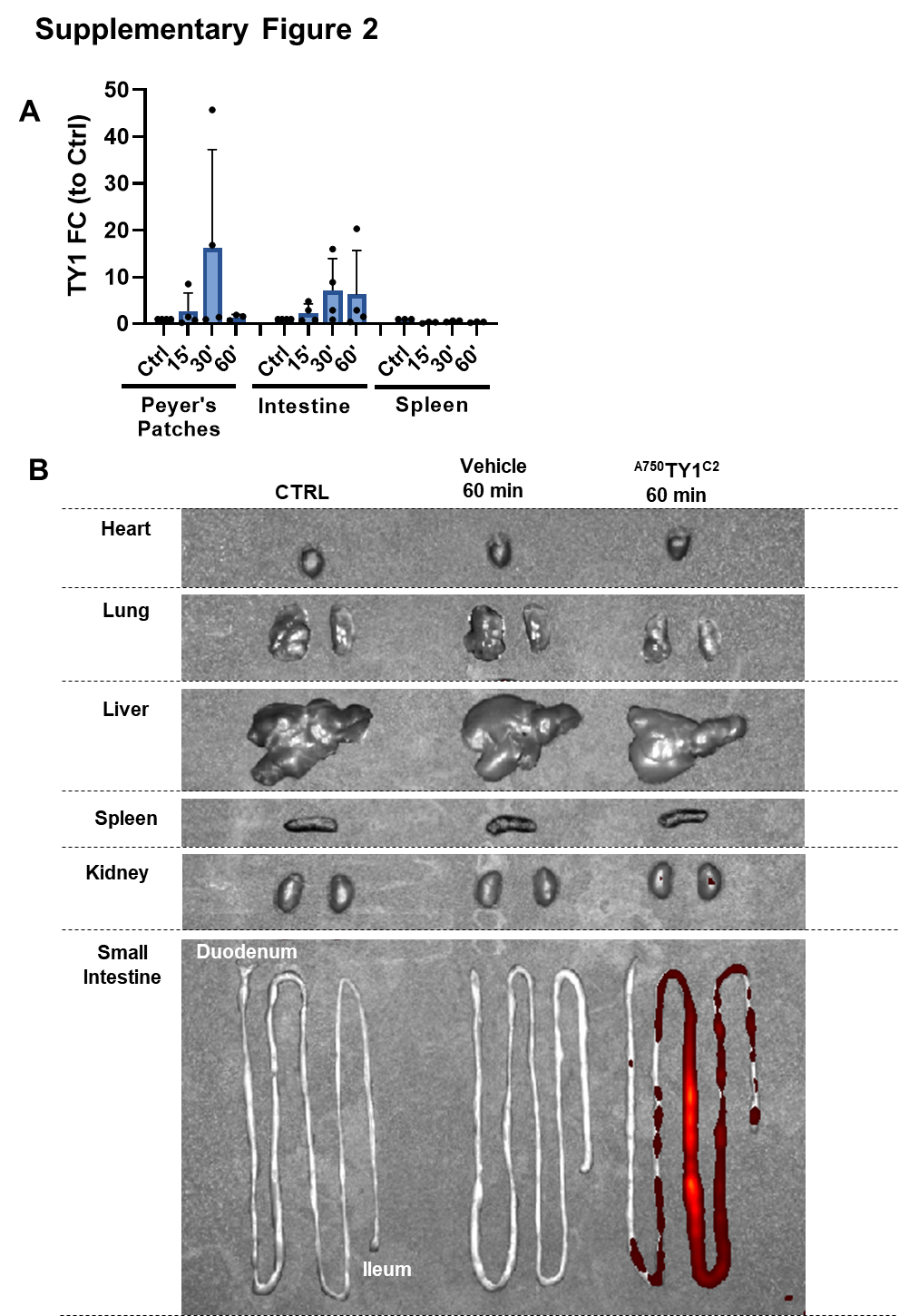


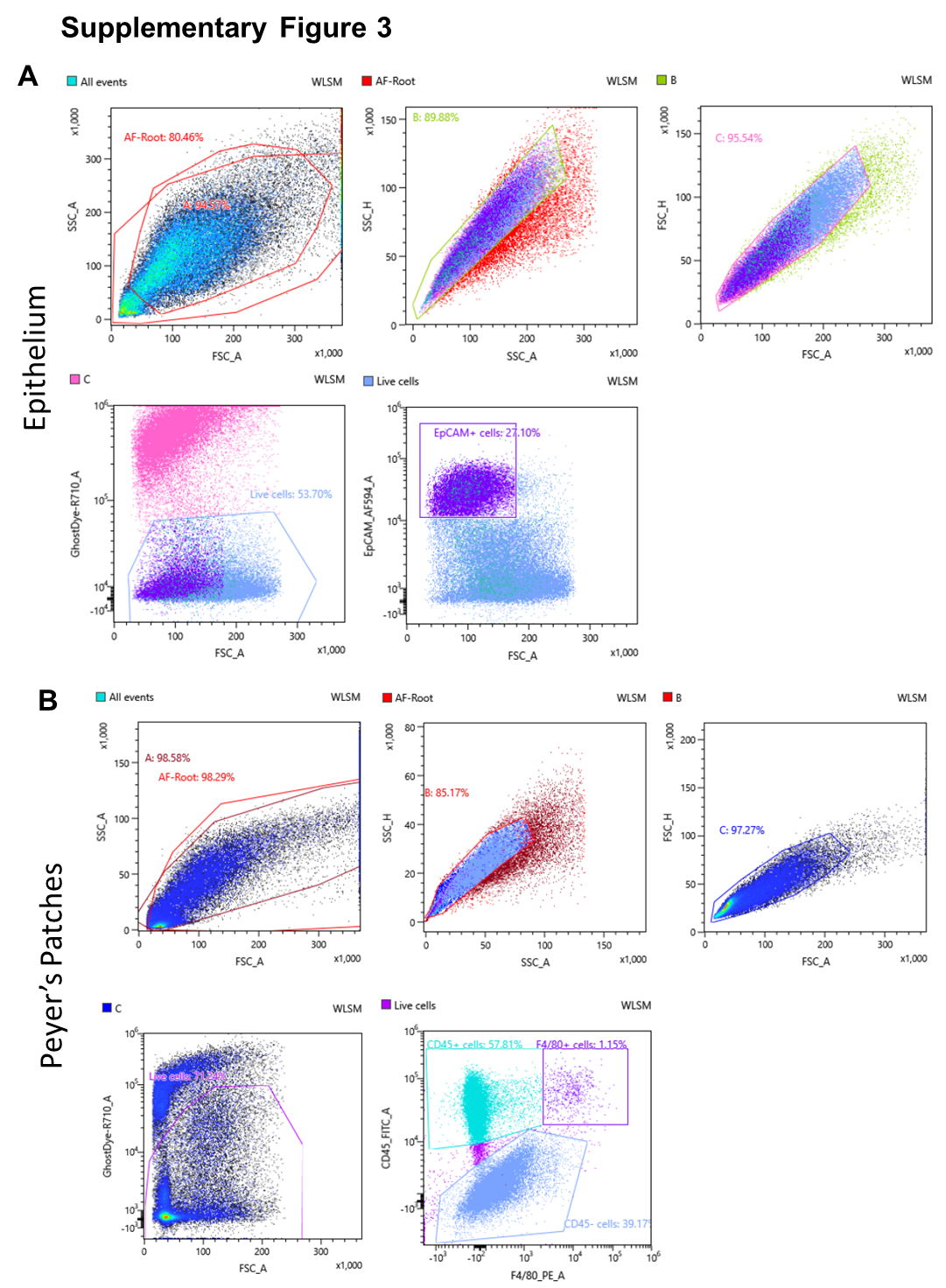


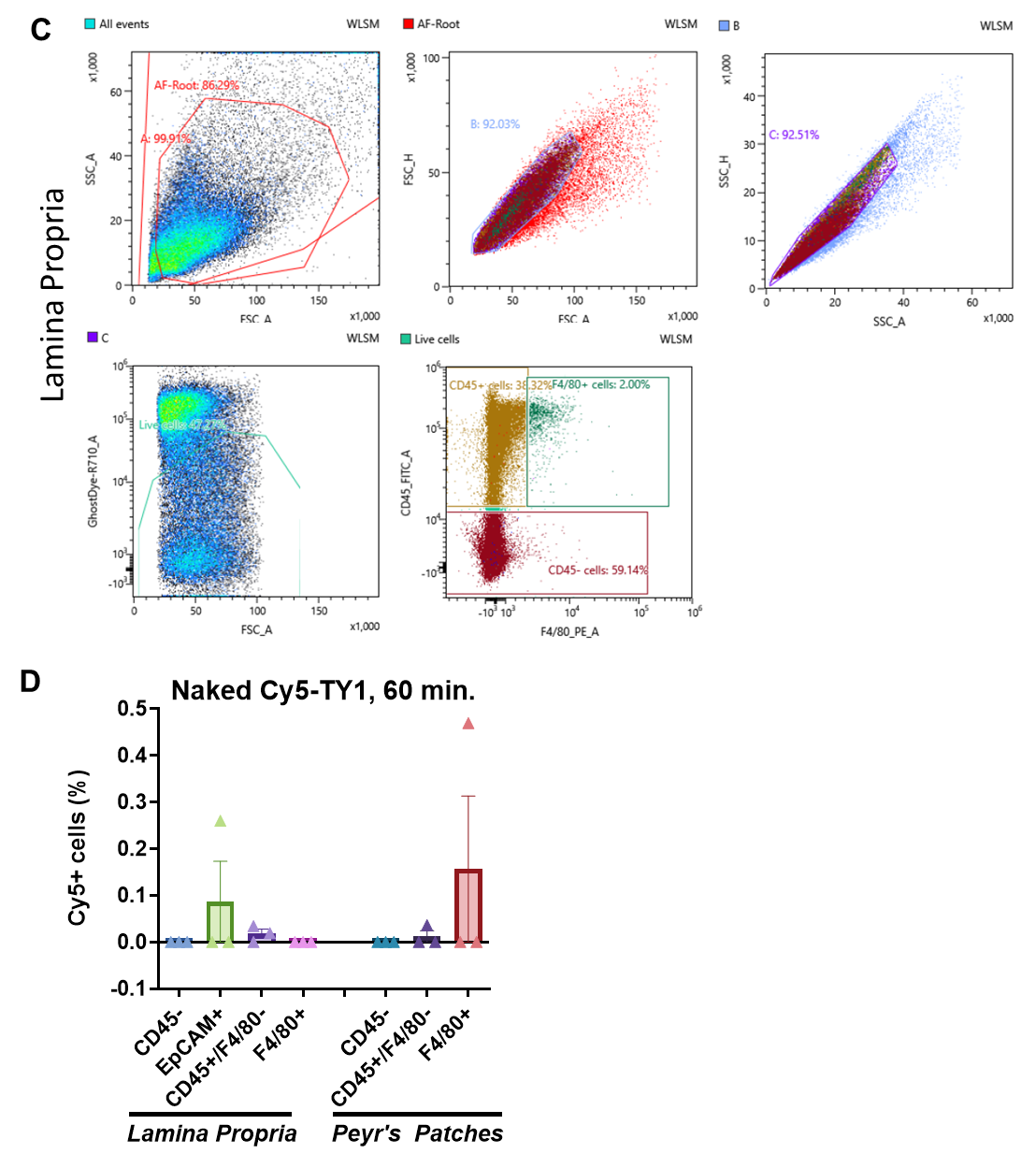


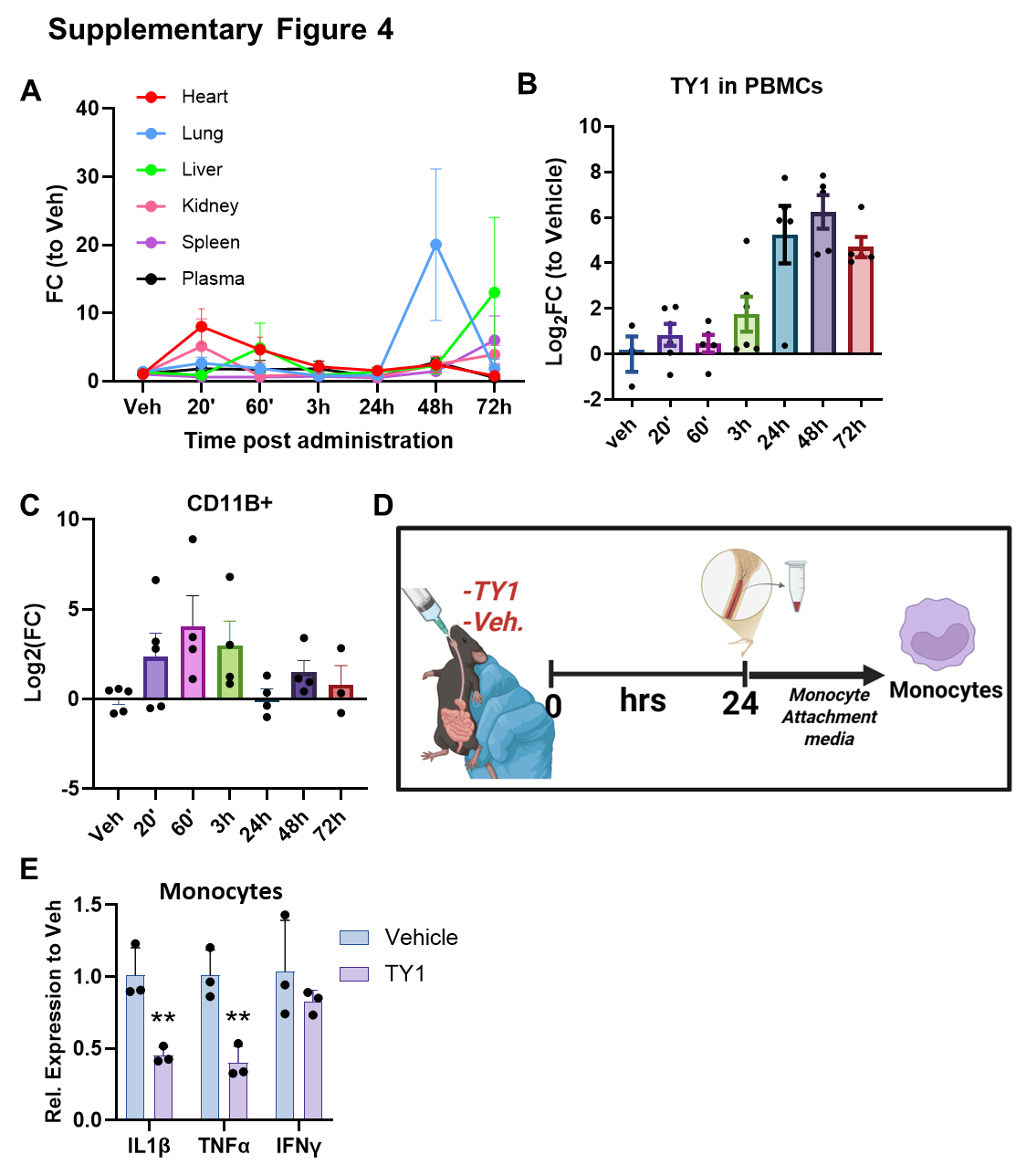

Supplement: Supplementary file 1 — Supplementary Table 1: Blood chemistries from animals dedicated to TY1 toxicity study. Metabolic panel of plasma samples from healthy animals that had been given vehicle, oral TY1 packaged in LNP only, TY1C, or TY1C2 twice a week for four weeks (n = 5 animals per group). Statistical Analysis was done using a One‐Way ANOVA with Tukey's post test to compare for multiple comparisons. Supplementary Figure 1: C2 formulation does not deliver small RNA cargo systemically. (A) qPCR demonstrated a lack of TY1 in organ tissues at 0, 20, 60, and 100 minutes post oral administration of TY1C2 (0.2 mg/kg). (B) TY1 was still undetectable even when the oral dose was increased 100‐fold (20 mg/kg). (C) Schematic for delivering intravenous and orally formulated siRNA to assess effects on liver tissue. (D) Successful suppression of Factor VII in liver tissue following intravenous or intraperitoneal administration of siRNA against Factor VII (siFVII). (E) Oral administration of C2‐formulated siFVII at higher doses of siFVII failed to suppress Factor VII in the liver. (F) Intravenous administration of siRNA against Gapdh (siGap) led to successful suppression of Gapdh in liver tissue. (G) Oral administration of C2‐formulated siGap failed to suppress Gapdh in both liver tissue and resident liver macrophages. Bars represent group means and error bars represent s.d. Supplementary Figure 2: TY1C2 biodistribution in mouse tissue. (A) qPCR of TY1 in Peyer's patches, intestinal tissue, and spleen demonstrating absorption of TY1C2 by Peyer's patches and (to a lesser extent) intestinal tissue (n = 4 animals per group). (B) Detectable fluorescence signal of A750TY1C2 in the mouse small intestine one hour post oral delivery with notable absence in other organs. Supplementary Figure 3: TY1C2 uptake by intestinal macrophages (A) Gating strategy for assessing uptake of Cy5TY1C2 in intestinal epithelial cells (B), Peyer's patches (C), and Lamina propria. (D) Animals fed Cy5‐labelled TY1 show impaired up [file JEX2-4-e70081-s001.docx]
